# Predicting non-attendance in hospital outpatient appointments using deep learning approach

**DOI:** 10.1080/20476965.2021.1924085

**Published:** 2021-05-24

**Authors:** M. Dashtban, Weizi Li

**Affiliations:** Informatics Research Centre, Henley Business School, University of Reading, Reading, UK

**Keywords:** No-show patients, deep learning, outpatient appointment, prediction, machine learning, health care, electronic patients records

## Abstract

The hospital outpatient non-attendance imposes a substantial financial burden on hospitals and roots in multiple diverse reasons. This research aims to build an advanced predictive model for predicting non-attendance regarding the whole spectrum of probable contributing factors to non-attendance that could be collated from heterogeneous sources including electronic patients records and external non-hospital data. We proposed a new non-attendance prediction model based on deep neural networks and machine learning models. The proposed approach works upon sparse stacked denoising autoencoders (SDAEs) to learn the underlying manifold of data and thereby compacting information and providing a better representation that can be utilised afterwards by other learning models as well. The proposed approach is evaluated over real hospital data and compared with several well-known and scalable machine learning models. The evaluation results reveal the proposed approach with softmax layer and logistic regression outperforms other methods in practice.

## 1. Introduction

Missed appointments have obvious operational and financial implications for health-care systems around the world resulting in health impact on patients’ groups who have unmet sufficient health needs (Ellis et al., 2017; Hasvold & Wootton, 2011). For example, from 2014 to 2015 only, there around 5.6 million (9% of the total) NHS outpatient appointments were missed in England (Quarterly Hospital Activity Data, 2019). Non-attendance can potentially lead to worse care for patients, inefficient use of staff, and increased waiting times. An estimate by the National Audit Office claimed that missed first outpatient appointments have costed the NHS up to £225 million in 2012 to 2013 (National Audit Office, 2014). Another estimate has placed the cost of missed UK general practice (GP; community-based family medicine) appointments at £150 million per year (George & Rubin, 2003). Recent Scottish government data suggest that each missed hospital outpatient appointment costs National Health Services (NHS) Scotland £120 (CampbelL. et al., 2015). Similarly, in the USA in a community hospital, it is reported that an average no-show rate of 62 appointments per day and an estimated annual cost of 3 USD million in a community hospital setting (Kheirkhah et al., 2015). It is also found that no-show and cancellation represented 31.1% of overall scheduled appointments among approximately 45,000 patients per year at a large family practice centre with an estimated total annual revenue shortfall of 3% to 14% (Moore et al., 2001).

Understanding the complexity of factors that contribute to non-attendance and predicting patients’ behaviours can develop targeted/personalised intervention to increase patient engagement and effective use of healthcare resources. Existing research on hospital non-attendance mainly focuses on finding associated factors in specific patient groups such as cardiovascular and diabetes. Other approach with additional attributes incorporating social economic, patient demographic and practice factors was proposed to investigate non-attendance patterns for general practices appointment in Scotland, but those variables were not analysed with data yet (Williamson et al., 2017). Although there are digital innovations developed for secondary hospitals to engage patients through mobile text message reminders, there is no evidence about what the reminder should contain in order to minimise missed appointments [2]. The key challenge is that there is scarce knowledge in pattern recognition and risk prediction of non-attendance in secondary hospital appointment. Moreover, patient behaviour and health usage problems result from a complex interplay of several forces. It includes behaviours, social environment, surrounding physical environments, as well as health care access and quality (Gerdtham & Johannesson, 2001). There are very few research studying the whole spectrum of big data incorporating those factors and their complexities for non-attendance prediction. One solution to utilise all of those factors is through deep learning. Deep learning as a particular subset of machine learning uses representation learning to map input features to output (Beaulieu-Jones et al., 2018) (analogues to prediction variables in traditional statistics). It learns latent features, non-linear relationships, and creates compact form of input features through several learning units (neurons) in many learning steps (epochs) (Goodfellow et al., 2016). This advantage makes the deep learning a preferable choice for many applications particularly where the data is high dimensional, sparse, or with many unknown relationships (Ferrão et al., 2020; Miotto et al., 2016). Beside the theoretical perspective, the key success of deep learning is its superior performance in many real-world applications (Georgevici & Terblanche, 2019; Miotto et al., 2016). Notwithstanding, deep learning is quite data-hungry such that it may not perform very well on small-scale data sets. This research aims to develop a novel approach to predict non-attendance based on deep learning on large healthcare-associated data (both in-hospital EPR data and outside-hospital data) with the following specific contributions:

1. ) predicting the risk of non-attendances for patients with future appointments, considering a large and highly diverse number of variables which can impact patients’ behaviour. Majority of existing research only identify non-attendance factors of certain diseases. Very few research developed the prediction model on an individual basis and usually include a limited number of factors;
2. ) developing a deep learning model based on sparse stacked denoising autoencoders (SDAE) to address representation challenges of high dimensionality, noise, sparseness, incompleteness, random errors, and imbalance in EPR. We adopt the SDAE for data reconstruction and prediction. Our model firstly learns the compact representation of data by which having missing values recovered, resulting in a better data representation. Then it uses a direct layer to predict the non-attendance event with an integrated softmax classification layer. Our approach is demonstrated to be more accurate based on performance evaluation with traditional machine learning methods in the context of outpatient appointment attendance.
3. ) risk profile with live patient data and intervention applications to reduce non-attendance. Different from existing machine learning where most of them still stay in performance experiments stage, we incorporate the prediction model into hospital information systems and public services for more targeted intervention and patient engagement.

This work is presented in several sections. The following sections present a review of previous works on non-attendance and deep learning studies in healthcare. The methodology in section 3 describes the training datasets, deep learning model and the training process. Section 4 presents the performance evaluation results, feature importance and model application in real hospital information systems. Finally, the conclusions and future work are discussed in section 5.

## 2. Literature review

Existing research on non-attendance mainly focuses on traditional quantitative and qualitative methods analysing factors and probability estimation for population groups. Most of the research in this domain studies factors contributing to non-attendance in both specific speciality and all appointments from the hospital or general practice. A variety of factors were found effective on patient’s attendance in paediatric urology unit (Bush et al., 2014), pulmonary rehabilitation (Hayton et al., 2013; Sabit et al., 2008), psychiatric (Killaspy et al., 2000; Mitchell & Selmes, 2007a, 2007b) and HIV (Catz et al., 1999), primary care (Giunta et al., 2013), inpatient and outpatient in the hospital (Shahriar Tavakoli-Tabasi, 2015) through analysing multiple correlation from hospital administrative database. A few studies also used survey and interviews to explore and compare the views of patient and health professionals on the reasons for non-attendance (Harte et al., 2018; Husain-Gambles et al., 2004; Lawson et al., 2005; Martin et al., 2005). The factors relate to inaccessibility, including physical location (Lasser et al., 2005), opening hours and days (Chariatte et al., 2007), and barriers such as language, stigma and cultural differences (Burns et al., 2007; Franks et al., 2007) may all be important. However, the interplay between the accessibility of a service and the perceived worthiness of the attendee, or “candidacy”, competing priorities (Harte et al., 2018; Mackenzie et al., 2013; Martin et al., 2005; Woods et al., 2005) (both self-perceived and as perceived by the service provider) can also lead to differences in how likely particular groups are to “get into, through and on” with services (Rosengard et al., 2007).

Moreover, morbidity differences can also affect attendance where the illness reduces the ability to navigate access to the health-care system (Mitchell & Selmes, 2007a). Variation in social and economic circumstances may mean certain times are inconvenient (Neal et al., 2005) and that the perceived importance of the appointment may vary between social groups in and of itself, or in the context of wider life complexities. Within psychiatry, for example, one study found that alcohol and drug users had particularly high non-attendance rates (Mitchell & Selmes, 2007a; CampbelL. et al., 2015).

However, above studies have focused on single disease areas. Studies of single disease area have produced conflicting results when it comes to designing effective interventions to reduce non-attendance (Cashman et al., 2004; Lehmann et al., 2007; Masuda et al., 2006; Nielsen et al., 2008). This may be due to a reliance on small data sets and limited variables in certain speciality settings. The non-attendance in primary care (Giunta et al., 2013), hospital inpatient and outpatient from all specialities (Shahriar Tavakoli-Tabasi, 2015) are studied focusing on single missed appointment. Factors are reported to be associated with age, sex, transport logistics, and clinic or practitioner factors such as booking efficiency and the rapport between staff and patients (Lawson et al., 2005; Martin et al., 2005; Murdock et al., 2002; Neal et al., 2005; Nielsen et al., 2008; Waller & Hodgkin, 2000). Williamson et al. (Williamson et al., 2017) and Ellis et al. (Ellis et al., 2017) focused on the patient demographics and practice factors that predict serial missed appointments in general practice. Although those studies considered multiple missing appointments as one of the factors, only a limited number of patient and practice variables. This has led to limited coverage of personal health, behavioural, environmental and social support information in the prediction model, lacking the capability of revealing the whole spectrum of patterns at the individual level. How the whole spectrum of patterns affects patients’ behaviour in attendance remains unclear.

Furthermore, those studies use population-based techniques rather than at an individual patient level. For example, logistic regression is mostly used to predict the probability of non-attendance by fitting numerical or categorical predictor variables in data to a logit function (Alaeddini et al., 2011; Ellis et al., 2017). The problem with these population-based methods is that they do not differentiate between the behaviours of individual persons and are based on small datasets. Therefore, it will affect the effectiveness of predicting results in practice. At present, little agreement exists on what works in practice to reduce missed appointments (Ellis et al., 2017).

Meanwhile, there is another group of research focusing on scheduling and rescheduling of no-show patients. The main aim of scheduling in healthcare system is to provide solutions to alleviate the problem of resource overburden, waiting room congestion, hospital-acquired infections, and longer appointment delays. Considering solely the outpatients appointment system, a practical strategy must cope with different events happening with appointment in rather real-time basis. Such approaches account for circumstances in which patients either come late, or cancel the appointment with a very short notice or won’t come at all. Although scheduling and rescheduling are not the focus of this study, its application in healthcare system specifically those adopted machine learning is quite relevant to ours in two perspectives. One perspective is that the variables they engage to build their models are somehow relevant and so could give us extra insight. Secondly, rescheduling is typically a post-hoc plan that can be added on top of predictive frameworks could increase practical effectiveness. Notwithstanding it is true that if a predictive system works rather well in a way that can be relied on, a rescheduling plan would be essential to increase the efficacy of system in practice. Meanwhile, this fact has been already accounted for in many hospital systems, each having their strategies to deal with cancelations, delayed patients, overbooking and no-show patients. To this context, some representative works are explained in the following.

Sharan and Ravi (Srinivas & Ravindran, 2018) developed a new rules-adapted framework for optimising outpatient appointment system using machine learning algorithms and scheduling rules. Their framework was basically motivated by a real-life case study involved in designing a real-time appointment scheduling system. This analytical framework employs machine learning to classify patients based on their no-show risk. This approach attempts to elucidate scheduling rules by incorporating three AS design decisions described such as no-show adjustment and patient sequencing. The identified rules are then assessed by considering the weighted sum of resource overflow time, resource overtime, resource idle time, patient waiting time and number of denied appointments. The main contribution of them lied in the development of eight novel appointment scheduling rules, which were modelled in the combination of sequencing and overbooking policies. They employed electronic health records and the variables such as patient information including age, gender, race, marital status, zip code, insurance group and weather data including minimum temperature, maximum temperature, and precipitation probability. However, they have not used geographical variables like the distance between patients’ home to the clinic which we employed too. Another successful application proposed by Samorani & LaGanga (2015) tries to develop an efficient overbooking strategy based on cost-sensitive Bayesian network and data mining techniques. Another representative work is in Deceuninck et al. (2018) where they developed a re-scheduling method for patients who do not attend or arrive late. Such scheduling and re-scheduling approaches can be quite useful in combination with accurate predictive solutions. In this research, our focus was on predictive modelling for which, briefly, a new deep learning approach is developed to consider a wide range of factors and extract important features and complexities towards meaningful patterns from the large dataset and more accurate at the individual level.

Actually, compared with traditional statistical methods, deep learning methods have attracted many researchers and institutions in clinical research tasks which are difficult or even impossible to solve with traditional methods (Raghupathi & Raghupathi, 2014; Wu et al., 2010). They are more robust to learn knowledge from high-dimensional and high-volume data such as health, social economics, and environmental information. It has proven to be competent to identify patterns and dependencies with cases superior to human experts. Therefore, deep learning methods provide great potential to present a whole picture embedded in large-scale data and reveal unknown structure to serve better prediction of non-attendance risk and effective engagement to optimise the health resource usage.

Deep learning classification from electronic patient records (EPR) is initially studied to predict disease progression. For example, (Choi et al., 2016) applied recurrent neural network (RNN) in longitudinal time stamped EPR to predict diagnoses and medications for the subsequent visit by building a generic temporal predictive model that covers observed medical conditions and medication uses, followed by the development of specific heart failure prediction model. (Pham et al., 2016) utilise the long-short memory (LSTM) method to model disease progression and predict future risk. Recently more attention is received in using deep learning method to predict the risk of readmission. For example, (Wickramasinghe, 2017) and (Wang et al., 2017) applied convolutional neural network methods to detect and combine predictive local clinical motifs to stratify the risk of readmission. (Jamei et al., 2017) developed an artificial neural network model to predict the all-cause risk of 30-day hospital readmission and (Xiao et al., 2018) developed a hybrid deep learning model that combines topic modelling and RNN to embed clinical concepts in short-term local context and long-term global context to predict readmission. (Rajkomar et al., 2018) further developed a scalable deep learning model using RNN for prediction across multiple centres without site-specific data harmonisation which is validated in readmission task.

However, as discussed, the existing application of deep learning in healthcare is mostly limited by the EPR data in the hospital. The tasks performed by existing deep learning research are highly clinical oriented such as disease detection/classification and sequential prediction of clinical events (Xiao et al., 2018). There is no deep learning research predicting patients’ behaviour while patients who miss appointments are more likely to have complex social and health needs (Husain-Gambles et al., 2004; Williamson et al., 2017). This research will contribute to the literature in developing deep learning methods that cover both EPR and outside hospital data to capture complex health and social situation and to predict patients’ behaviours.

## 3. Methodology

**Figure 1.**
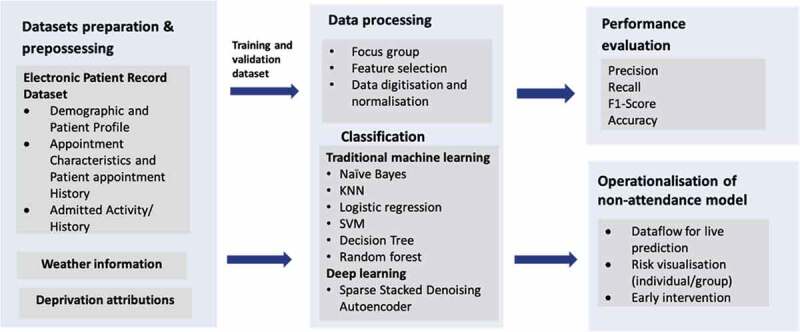
Research framework to develop non-attendance prediction model and evaluating performance gains from deep learning architecture.

This section introduces our methodology from datasets preparation, classification model, performance evaluation to the operationalisation of non-attendance prediction model as shown in Figure 1. The proposed deep learning approach is actually an end-to-end model that starts from pipelining the data through to prediction stage and presentation layer which is actually the outpatient management system in hospital. Similar to any other data-driven machine learning application in practice, this approach is typically comprised of several stages including data acquisition, processing to model development and deployment.

In the following section, first this deep learning approach is more technically detailed in two subsections involving the theoretical foundations behind SDAE and its training phase. Then, in the other section, the description of data, processing and balancing techniques are detailed. Finally, the performance of proposed approach has been compared with other well-known methods.

### 3.1. Deep learning model based on sparse stacked denoising autoencoders (SDAE)

Hospital information systems typically process high-dimensional EPR data. Moreover, they store data where attributes have a large number of missing values (Miotto et al., 2016). There are several algorithms in the literature to deal with such issues. The simplest way is to replace the missing values with the mean values, median values, or some other statistics. It is naturally fast and straightforward but not effective as it does not include the relations of such missing values with other known/unknown values. To this point, the SDAE is an unsupervised learning solution for reconstructing the whole data through by recovering the missing values and provide a compact data representation. Additionally, learning highly non-linear and complicated patterns such as the relations among input features is one of the prominent characteristics of SDAE (Suk et al., 2015). To this end, in this paper, the SDAE was employed for recovering whole data in the first step (after data preparation from our hospital EPR system).

**Figure 2.**
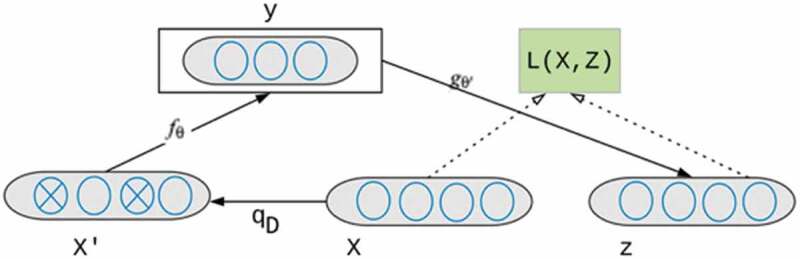
Denoising Autoencoder Architecture.

A denoising autoencoder (DAE), as shown in Figure 2, is a neural network with one hidden layer that should be trained to reconstruct a clean version of input X from a corrupted/current version of x’ through a stochastic mapping $\tilde{x}q_{D}\left(\tilde{x}|x\right)$. It is accomplished by a so-called encoder that is a deterministic mapping from an input vector x into hidden representation y. X is a dataset with variables to predict patient’s non-attendance mentioned in section 3.1.

$$f_{\theta }\left(x\right)=s\left(Wx+b\right)$$

where the parameter θ is (W, b), W is a weight matrix indicating the weight of each of the contributing variables of patients with non-attendance, b is an encoding bias vector. In denoising autoencoders, the loss function is used to minimise the reconstruction loss between a clean X and its reconstruction from Y [50]. A decoder is then used to map the latent representation into a reconstructed (“repaired”) vector such as z∈ [0,1]^d where W’ is a decoding matrix, and b’ is decoding bias vector;

$$z=g\theta \;\prime \left(y\right)=sWy+b$$

In stacking denoising autoencoder (SDAE), the auto-encoder layers are placed on top of each other. Each layer is trained independently (“greedily”) and then is stacked on top of the previous one. The SDAE could have several layers. For training an SDAE, each layer is trained on top of the previous one. The training process starts with pre-training the first hidden layer fed the training samples as input, training the second hidden layer with the outputs flowing from the first hidden layer and so on. This is how autoencoders stack hierarchically to form a deep SDAE. The parameters of the model θ and θ′ are optimised during the training phase to minimise the average reconstruction error,

$$\theta ,\theta^{\prime *}=arg\underset{\theta ,\theta^{\prime *}}{\min}L\left(x,z\right)=\arg\underset{\theta ,\theta^{\prime *}}{min}\frac{1}{N}\overset{N}{\underset{i=1}{\sum }}L\left(x^{\left(i\right)},z^{\left(i\right)}\right),$$

where L(x,z) is a loss function, and N is the number of data samples in the training set. The reconstruction cross-entropy function is usually used as the loss as depicted in the equation below:

$$L_{H}\left(x,z\right)=-\overset{d}{\underset{k=1}{\sum }}\left[x_{k}logz_{k}+\left(1-x_{k}\right)log\left(1-z_{k}\right)\right]$$

One serious issue concerning autoencoders is the size of the hidden layer that could potentially affect the performance. If the dimensionality of the hidden unit (number of neurons) is the same as or larger than the input layer, this approach could potentially learn the identity function. It means that the model would overfit to input data instead of learning non-linear relations. Furthermore, employing larger dimensionality conducts the model to learn a sparse representation of data which may result in learning more latent variables and non-linear relations. Considering to use the denoising type only may ultimately result in learning the identity function, whereas (Xie et al., 2012) showed that sparse type of denoising autoencoders could learn other features than the denoising type. In this regard, espousing a sparsity constraint could practically solve such issues providing SDAEs with more hidden units of larger dimensionality. The equation below depicts a sparsity constraint added to the previous equation.

$$SC=\;L\left(X,Z\right)+\gamma \overset{H}{\underset{h=1}{\sum }}KL(\rho ||{\hat{\rho }}_{j})$$

where γ denotes the weight of penalty factor, H is the number of hidden units, ρ is a sparsity parameter and is typically a small value close to zero, ${\hat{\rho }}_{j}$ is the average activation value of hidden unit j over the training set, $KL(\rho ||{\hat{\rho }}_{j})$ is the Kullback–Leibler (KL) divergence as defined below.

$$KL(\rho ||{\hat{\rho }}_{j})=\;\rho \log\rho \;/\;{\hat{\rho }}_{j}+\left(1-\rho \right)\log\left[\left(1-\rho \right)/\left(1-{\hat{\rho }}_{j}\right)\right]$$

The KL is principally an asymmetric measure of the distance between two given sample distributions. It provides the sparsity constraint on the coding. For instance, if two distributions are equal (e.g., ρ=${\hat{\rho }}_{j}$), the KL would be zero. A standard backpropagation algorithm can be used to solve this optimisation problem.

Besides data recovery and construction by non-linear transformation resulting ultimately in a compact representation, the SDAEs could include a standard predictor to make the predictions. This layer could be a proper function like logistic regression, max and softmax. In this work, we used a softmax layer which has proven performance in the most recent application. We will predict not only binary classification but also more detailed patients’ attendance behaviours including attendance, non-attendance without prior notification and non-attendance with prior notification through multi-classification as the next step future research. Furthermore, using softmax will get a probability distribution which we can apply cross-entropy loss function. This layer contains a softmax function as depicted below.

$$p_{\left(y=j|x\right)}=\frac{e^{x_{j}}}{\sum_{k=1}^{N}e^{x_{k}}}$$

where x is an N-dimensional vector of real numbers from the previously hidden unit and transform it into a vector of a real number in the range $\left(0,1\right)$ thus, it is the output probabilities for each class. As is clear in the equation, the output is always positive numbers which have also been normalised.

### 3.2. Model training

For training the model, the conventional practice was followed such that 75% of data over time was employed for training data. The remaining records were utilised as testing data for evaluating the model performance. We tried to use a natural split as the model is going to be run over the live data, the most recent data samples were used for testing the model comprising statistically around 25% of all samples. The remaining samples were divided using stratified random sampling into of 15% validation and 85% training sets. In this context, it is worth noting that the conventional split in data science practice is 70–30% train-test split from which, a small proportion of testing samples were drawn for model selection. However, in our evaluations for model selection, other splitting odds including 1:9, 5:5 were additionally experimented. Nevertheless, those splits did not reveal any better performance.

For model selection part, the Stratified random sampling (Marqués et al., 2013) is essential to maintain the original class distribution among both subsets. Moreover, in stratified random sampling, all features were used to select more balanced subset for validation (model selection) purpose. Furthermore, a simple random sampling could also be employed but may not guarantee to have an equal ratio from two classes whereas we will need them to select a model that generalises upon both two classes.

In brief, the training model is able to minimise the difference between the feeding data and recovered replicate (i.e., the output of the autoencoders) while trying to build an overall high-performance classification model with backpropagation. It is noteworthy that the pre-training the SDAE layers is unsupervised as no label is being used. However, the optimisation process is supervised as we exploit the target vector (i.e., prepared binary labels indicating attendance vs. non-attendance). Our method was implemented and evaluated with SQL Server (for fetching data, preparing tables and cleansing), Matlab 2018a (deep learning and machine learning packages) and Jupyter Notebook. The experiments were conducted on CPU 4 Ghz, RAM 32GB, Highest Speed SSD: 1TB, and VGA Card: GTX 1080TI with 11GB of RAM having over 3600 CUDA cores.

The training of the model comprises two phases. At first, the model is trained using a training dataset together with its associated labels. In the former phase, we try to minimise the difference between the recovered and ground truth training dataset: X vs. X. In the later phase, the purpose is to optimise the model regarding supervised prediction performance.

It is worth mentioning that training the model using standard backpropagation algorithms usually yields poor performance. To this end, a greedy layer-wise unsupervised learning algorithm is proposed by (Hinton et al., 2006) to pre-train the SDAEs layer by layer in a bottom-up way. Just afterwards, fine-tuning the model’s parameters in a top-down direction is applied with backpropagation to improve the performance at the same time. The training procedures of this study briefly involve the following steps drawn from the proposed algorithms in Bengio et al., (2007) and Hinton et al. (2006).

**Step 1**: Minimize the objective function of the first autoencoder over the input data

**Step 2**: Minimize the second autoencoder’s objective function over the output of the previous layer

**Step 3**: Iterates through steps 1 and 2

**Step 4**: Obtain the probability of no-show patient class based on the output of the last hidden layer

**Step 5**: Optimize the whole network with backpropagation algorithms

The first three steps are unsupervised as it is aimed to minimise the reconstruction error; whereas in the last step, where the generated labels from the last autoencoder fed to a softmax layer, all stacked layers will be optimised using backpropagation as a whole network. The optimisation is performed in a supervised way based on the respective class labels.

Moreover, it is critical to consider that the number of hidden layers could potentially leverage the performance of SDAE. Very shallow structure of SDAE could result in poor performance whereas a very deep structure (i.e., with many hidden units) makes the constructed model very complex and diversely affects the performance as well. We used a three-layer SDAE according to classification experiment from 0 to 5 layers using training and validation data sets. The Area of Under Curve (AUC-ROC) and F score stabilise after using three-layer SDAE. The shallower networks resulted in poorer performance have failed to learn proper representation while going very deeper added just complexity than any improvement. Our empirical observation was already reported in (Vincent et al., 2010) as they also found the higher stability of results (error convergence) on the three-layer architecture, especially for sparse types.

## 4. Experimental analysis

This section compares the performance of traditional classifiers and deep learning architectures in predicting outpatient appointment attendance. The evaluation provides evidence that deep learning is superior to traditional classification approaches in predicting patients’ attendance behaviours.

### 4.1. Dataset

**Figure 3.**
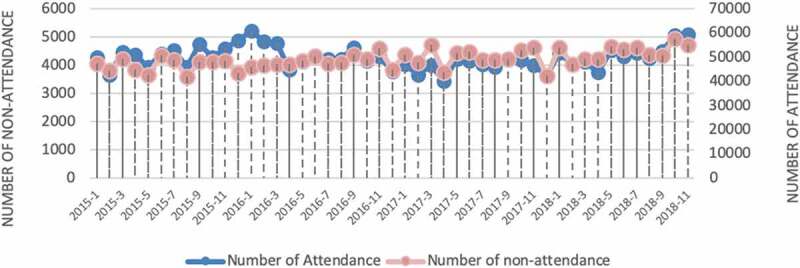
Number of attendances vs non-attendances during 2015 to 2018 at monthly basis (note two stacked lines follow different scales given at vertical axis in right and left of the figure, respectively).

The data source is from in-hospital data (e.g., electronic patient records (EPR)) and outside hospital data (e.g., environmental and social, economic data). In EPR, the information of over 150,000 outpatients spanning on around 1.6 million records were gathered from an acute NHS hospital in the UK. The information is distributed beginning from April 2015 and going through September 2018. Figure 3 demonstrates the number of attended appointments and non-attendance appointments each month. The number of attended appointments varies from 42,008 to 57,581 while the number of non-attendances varies from 3450 to 5230. The total number of non-attendance appointment records is 298,812, and the total number of appointments is 3,747,285.

As shown in Table 1, the model variables cover various areas that could affect attendances including demographic and patient profile, appointment characteristics and patient appointment history, deprivation attributes, weather and activities carried out after the patients’ admission. Those variables are identified through literature and focus groups with hospital operation teams who manage appointments at the frontline, which represent domain knowledge. In line with what was found in the literature, with several studies proving the added value of integrating the domain knowledge into forecasting models, these five groups of variables will be considered for the construction of the non-attendance models. A complete list of variables is added to the Appendix 1 for more reference.

**Table 1.** Brief description of variable groups for non-attendances prediction.

CATEGORY	VARIABLE
Demographic	Age, Gender, Ethnicity,
Patient History	Multi-Comorbidities, Address stability
Appointment Characteristics	Follow-up or first-time appointment, GP referral time to appointment, care Speciality, Site
Time Variables	Day, month, year, time of the day
Patient Appointment History	Statistics on number/ratio of attended and non-attended within and out of 30 days,
Socioeconomics	Education decile, Index of Multiple Deprivation, Income Decile, Living Environment Rank, etc.
Weather	Temperature, Condition (e.g., rain/snow, etc.), Humidity
Admission History	Recent admission, length of Stay, Procedure, time interval

Regarding Table 2, our dataset contains not only outpatient information but also inpatient information. We used it to take the advantages of possibly available historical health data when new-coming patients had previous in-patient experiences. Such historical health records contain diagnostic codes which in turn could be used to draw some very informative variables from the patient profile such as co-morbidities. If a patient had inpatient records for more than once, we will only use the record where there was an overlap between inpatient period and outpatient appointment time or less than 14 days gap between discharge and outpatient date. This is based on the discussion with focus groups that patient may choose not to attend the outpatient appointment if it is within their inpatient time or it is close to their discharge date. It should be noted that some variables are particularly conditional. For instance, length of stay (LOS) is non-zero if and only if the patient had an immediate inpatient record in the EPR. The zero value is used for every empty element in the resulting table if the patient did not have an immediate inpatient record.

**Table 2.** Distribution of non-attendance over ethnicity group, care group and gender type.

Attribute cluster	Levels	Non-attendance	total appointments%
**Ethnicity**	British	62.39%	68.23%
	Non-British	20.57%	16.98%
	Not Known	17.04%	14.79%
**Care Group**	CG2 – Planned	52.65%	52.63%
	CG3 – Networked	26.71%	28.21%
	CG1 – Urgent/etc.	18.97%	19.17%
**Gender**	Female	55.40%	57.6%
	Male	44.69%	42.4%

Moreover, each variable has statistically or intuitively its own association with non-attendance event as addressed in the literature and empirical data from hospital focus group. The deep learning-based methods involve the contribution of all variables to the target model rather than considering each singularly. We have three kinds of variables comprising categorical variables, nominal variables, and real-valued types. For the first two types, we performed digitisation in which, distinct values of each variable were extracted, and a unique number was assigned to. After digitisation, a normalisation procedure was applied to centre the data and making them in a closed range [0,1]. The normalisation considerably diminishes the inverse effect of large-scale variables to hinder the network from incorporating small-scale attribute in both the neural networks and classification models (Witten & Frank, 2002). Besides the input variables, the target variable that is a binary event i.e., attendance & non-attendance, should be constructed. The target vector contains either zero or one for the corresponding event for each row of information.

Furthermore, considering Table 2, some variables could be merged to create representative variables such as these deprivation indexes. However, deep learning attempts to learn the relations and high-level representation of variables (Goodfellow et al., 2016), thereby making the feature engineering phase easier. On the other hand, sometimes ignoring some variables may reversely affect the final model as we do not know some hidden relations which laid within data. Table 2 represents some of those important variables.

This Table depicts the distribution of non-attendance with respect to ethnicity, care groups and gender over all the data in this study. As can be seen, most of the care groups are planned (over 52%) and the majority of non-attendance coming from British (62.4%). This, in turn, can be an indicative for machine learning classifier to predict the non-attendance; nevertheless, no conclusion can be literally drawn; since the non-attendance is dramatically higher for British ethnicity as most of the appointments concern them as well. For male group, similarly, higher proportion of attendance comprises females and they have higher proportion of non-attendance in the cohort.

**Figure 4.**
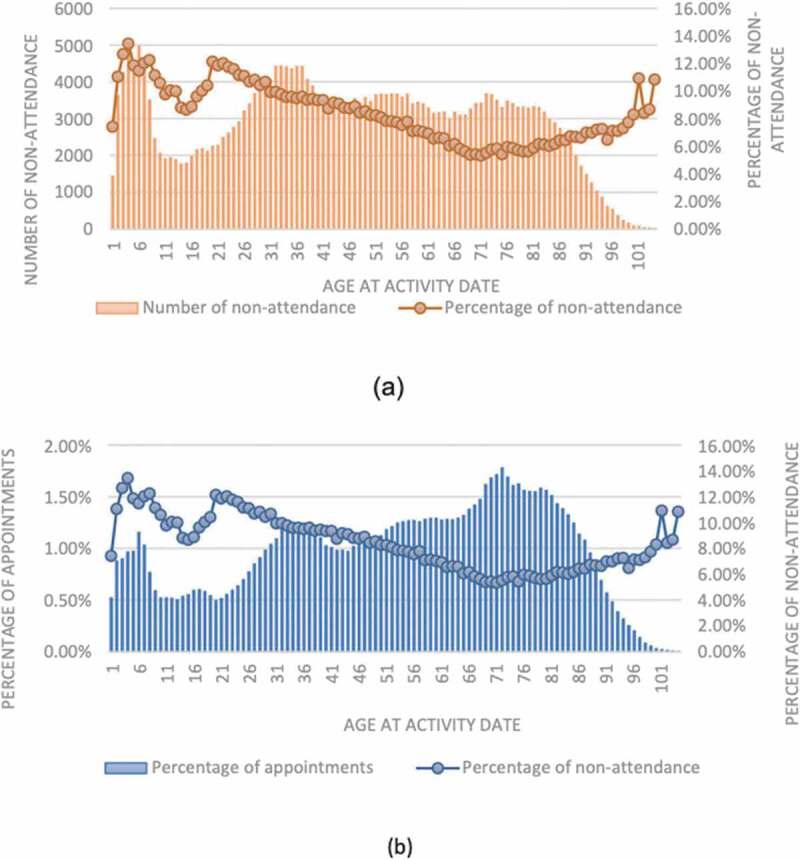
Trend of non-attendance at different ages: (a) exhibits the number of non-attendance against the percentage of non-attendance at each age group, and (b) demonstrates the percentage of non-attendance vs the percentage of appointments.

Furthermore, the distribution of non-attendance across different age groups is demonstrated in Figure 4. As can be seen, the non-attendance rate varies between roughly 6–13% within different age groups. Besides, the highest proportion of non-attendance is coming from ages 2–10 and 22–28-year-old patients all having non-attendance rate greater than 11% (Figure 4(a)). Nevertheless, the higher proportion of appointments have actually booked by elderly patients looking at Figure 4(a). This fact is more evident particularly where the total number of appointments starts sharply increasing at age 43 reaching its peak at 73; nonetheless, the degree of non-attendance faces a consistent decline at the same time interval. This fact in our data actually states the elderly patients who required evidently more provision of care utilises services more efficiently having lower operational burdens in practice. There could be many discussions around which is basically out of the scope of this paper.

Besides these figures, the distribution of non-attendance as well as other statistics, are all provided in Appendix A. In the current practice, descriptions of these variables are reported to hospital operational team monthly to understand the characteristics of patients with non-attendance. However, a more accurate prediction of non-attendance risk on an individual basis from large data set is needed for the operation team to contact the high-risk patient, which will be discussed in the following sections.

### 4.2. Class imbalance

One central challenge in many real-life applications is class imbalance ignoring which results in over-classifying the majority group due to its increased prior probability (Johnson & Khoshgoftaar, 2019). There are many types of class imbalance as discussed comprehensively in this review (He & Garcia, 2009; Manonmani & Balakrishnan, 2020); concerning intrinsic and extrinsic properties, “Variable factors such as time and storage also give rise to data sets that are imbalanced”. The time property is an important factor within in-hospital data casing it to be relatively highly imbalanced. For example, a patient may have even 500 appointments in 3 years while one may have a few one. Therefore, the data induces two critical challenges. First, the data is biased in favour of patients with significantly more records. Subsequently, it can be said that the data is biased at individual level. Secondly, the rough probability of outcome of the interest is about 7% in our hospital. It denotes a very imbalance odd of 93:7 revealing a quite high imbalance. There are several rather successful method in literature targeting class imbalance mostly through resampling techniques such as what is listed in this survey (Johnson & Khoshgoftaar, 2019). Or, using ensemble learning techniques and classifiers such as Random Forest or XGBoost (the newer version of Random forest with lower time complexity). Among such resampling techniques we can name Random Under Sampling (RUS), Random Over Sampling (ROS), the combination of both RUS/ROS, SMOTE-Synthetic Minority Oversampling Technique (Johnson & Khoshgoftaar, 2019) and other hybrid techniques. However, most of them endeavour mainly to randomly create target-based balanced sets; actually, ignoring the fact how such imbalance was created. Thus, in some application, it is wise to seek other way around to see if we can do something about the data itself. Hence, in this study, a simple yet effective strategy to deal with both challenges of class imbalance and high individual-level bias was adopted by simply removing consecutive successful appointments at care speciality level. Thus, on the one hand, this process reduces the individual bias and on the other hand, led to significantly more balanced sets. Regarding the proposed strategy for class imbalance, several small-scale experiments on bootstraps of data have been conducted employing different strategies. Ultimately, it was found that a few strategies work superior as below:

(a) Removing successful follow-ups,

(b) Removing repetitive appointments,

(c) Removing repetitive appointments at treatment speciality level,

(d) Removing successful follow-ups at treatment speciality level.

Additionally, we found that the cancelations’ records work typically like noise for this application. Thus, they should have been actually removed both in training and testing phase. It was operationally intuitive; though it was not noticed beforehand. That is actually a true fact that when an appointment is cancelled, literally there is no appointment at all to predict or not; thus, why it should have been involved for prediction. Despite that, predicting cancelation or particularly late cancelation can be quite interesting which is out of the scope of this research. Among all these four strategies for balancing, as can be noted in the above algorithm, strategy (c) unveiled to have higher performance while producing more stable results in many small-scale experiments.

### 4.3. Evaluation

The evaluation phase consists of three stages. In the first stage, the original test data was fed into the previously trained model. The trained model will elucidate the recovered version of the feeding test data while at the same time producing a probability of non-attendance event. There are multiple evaluation measures in the literature to evaluate the performance of a predictive model. However, the most important ones in practice specially when the data is highly imbalanced are precision, recall, and F-score. Recall is actually the detection rate indicating the capability of a predictive model to identify positive class of interest (non-attendance patients in this study). It is obvious that the higher a system can detect such cases the higher capacity we have to prevent such events happening. On the other hand, high. Number of false alarms can potentially prevent taking any interventions particularly for big data application. Because, we do not have either huge resource or that might cost more than it benefits. Thus, the precision of a predictive model plays a very important role which is actually an indicative of proportion of false alarms too. Taking both into considerations, F-measure was introduced in literature to provide a weighted measure of combination of both precision and recall which is computed using the following formula.

$$F-measure=\frac{2\times Precision\times Recall}{Precision+Recall}$$

Considering F-measure, one can say, a system with higher F-measure is probably a better system. Nevertheless, it is not always true. Assume that we want to use that such models in practice like our application. Then, it is sometimes better to slightly compromise F-measure for higher recall.

The proposed method was applied to the test data and its performance compared with representative predictive methods presented in Table 3. All these methods have been evaluated over the balanced data since almost all of them failed to generalise over imbalanced original data. Seven well-known machine learning classifiers were employed and benchmarked including support vector machines (SVM), K-nearest neighbours’ algorithm, Decision Tree (DT), Naïve Bayes, Random Forest, Rotation Forest (Rodriguez et al., 2006), Logistic Regression (Hilbe, 2009). Many of these methods have already shown promising results in different and similar areas of healthcare [1]. In this study, we utilised these methods with different parameter settings too. In experimentations for approximating near optimal parameter settings, aside from guidelines from previous studies over high-dimensional data such as previous studies (Dashtban & Balafar, 2017; Dashtban et al., 2018; Duda et al., 2012; Wang et al., 2018), we applied these methods upon some bootstraps of data. Applying such methods on whole data is quite resource intensive although we have already had quite great resources in place provided by NHS Foundation Trust. In this context, using other guidelines, previous experience, and bootstrapping could save a lot of time and resource. Thus, we vary the hyperparameters of those methods to see if we can see a significant change. If so, we further continued diving through. In this regard, Table 3 represents the performance of classifiers over whole data only with few important hyperparameters that rather reveal some changes.

**Table 3.** Performance of different predictive methods (*numbers in bold represent top five F1-scores over 0.21).

Method	Measures		
	Precision	Recall	F1-Score
Logistic Regression (Hilbe, 2009)	0.197	0.286	0.233
SVM + Linear	0.115	0.487	0.186
SVM + Polynomial = 3	0.122	0.557	0.200
SVM-RBF Kernel	0.094	0.617	0.163
KNN (best K = 50)	0.059	0.926	0.111
KNN (best K = 3)	0.062	0.941	0.117
Naïve Bayes (Kernel)	0.146	0.424	0.217
Naïve Bayes (Normal)	0.200	0.143	0.167
Bayesian Network Classifier	0.175	0.272	0.213
Decision Tree (Optimised, pruned, min leaf = 2)	0.101	0.451	0.165
Random Forest (optimised,2000 trees, 50 cycles, minleaf = 10)	0.176	0.415	0.247
Rotation Forest (K = 10)	0.117	0.514	0.191
Rotation Forest (K = 50)	0.081	0.751	0.146

Moreover, hyperparameters vary with different methods or classifiers. For example, for KNN classifier the main parameter is K that is an indicative of number of nearest patients to the case we are going to decide on. K should be usually an odd number. The higher the K, the more time-consuming the learning carries out. It seems for our application, higher K resulted in worst performance. It is highly possible. Because when K is larger, more uncertainty imposed from more patients would be accounted for and more probably the generalisability of KNN classifier becomes lower. Nevertheless, the performance difference is not that significant at all; less than 0.02 in all the three measures (recall changes from 0.92 to 0.94, likewise for F-measure).

Naïve Bayes classifier that learns based upon Bayes theorem is inherently a probabilistic approach. Similar to other probabilistic approach like Bayesian Networks works basically on the grounds of normal distribution by default. Nonetheless, its performance is remarkably higher with Kernel distribution (with F-measures of 0.16 and 0.21 for normal and kernel distribution, respectively; Naïve Bayes classifier with Normal distribution leads to higher precision model (0.20 vs 0.14) though). Kernel distribution is realised by statisticians to work superiorly over skewed distribution that is actually the case of many high-dimensional data (Dashtban et al., 2018).

The performance of Bayesian Networks (BNs) was assessed using the Bayes Net library implemented in Weka Data mining package which actually adopts Hill climbing search and Bayes simple estimator. There are other estimators such as taboo search, genetic algorithm and other search strategies for which their time complexity is expansional and consequently not applicable for large data samples. BNs are actually the extension of Naïve Bayes and accordingly employ probabilistic approach for prediction. They do not involve representation learning which is intrinsically included in Deep Learning methods. On the other hand, for high-dimensional data (data with many features), the deep learning is the one that has unveiled many successful applications in different domains. We applied this approach to our data and the results reveal no better performance than Naïve Bayes classifier. One possible reason is possibly that Bayesian Network approach may work much better on an individual level rather than all data. Another speculation could be the representation of data was not very suitable for Bayesian learning. Utilising probabilistic approach in more comprehensive way could be a potential future work.

Support Vector Machine (SVM) classifier which is a popular supervised classification method been widely successful in many complicated classification tasks such as cancer diagnosis (Huang et al., 2018). SVM can be used with different kernel functions to learn linear and non-linear relationships in database feature space by forming different hyperplane decision boundary between the classes. There are many kernel functions associated with classification and prediction that are nicely described by Yuichi Motai (Motai, 2015). Among them, three most widely used kernels with SVM are Gaussian Radial Basis function (RBF), Linear Kernel and polynomial kernels. Linear kernel can be defined as a polynomial function of degree 1. The time complexity of RBF and Polynomial is way higher than linear kernel function. However, in this application, SVM with polynomial function of degree 3 has a relatively higher F-measure of 0.20 followed by linear and RBF kernels with 0.18 and 0.16, respectively. With RBF, the SVM reached its highest recall rate of 0.61 though having the lowest precision of 0.09 among other kernels. However, its F-measure is the lowest; one interesting point is that the fact that when KNN classifier does not function very well over various K, the SVM with RBF does not work very well too. There are actually some theoretical foundations for that too; as both KNN and RBF are non-parametric methods that estimate the density of probability of different regions in feature space. Nevertheless, that does not strictly state that the performance of one is an indicative of the other. Overall, it can be said that SVM performed as good as Random Forest and Probabilistic approach like Bayesian Network reaching to F-measure of about 0.20.

Decision tree-based classifiers have added benefit over other classifiers as they can work directly over categorical variables making them more suitable for real-life application with various types of features. That is possibly why they have been mostly successful in big data so far (Genuer et al., 2017); having both sequential and even parallel implementation for distributed computing (Chen et al., 2017). Decision Tree, itself, is among the simplest widely used baseline method with relatively competitive performance. “The key advantage of decision trees over other methods is that they are very interpretable, and in many applications, such as healthcare, this interpretability is often preferred over other methods that may have higher accuracy but are relatively uninterpretable” (Bertsimas & Dunn, 2017). Random forest (RF)generally performs better for more complicated classification task particularly when the number of variables is much larger than the number of samples (Biau & Scornet, 2016). The RF classifier is basically on the basis of creating several randomised decision trees and aggregates their predictions by averaging. Random forests can be thought of as a multi-agent system that functions better when there is high uncertainty in data. The experimental results exhibit a significantly higher performance of Random Forest over all other classifiers followed by Logistic Regression with F-measure of 0.24 vs 0.23 for RF and Logistic Regression, respectively. However, the model generated by Random Forest is a more practical solution. Because the recall of Random Forest is markedly higher than that of logistic regression (recall of 0.41 vs 0.28) whilst the difference in precision between the two is only 0.02 (0.17 vs 0.19). Considering other factors, it is necessary to note that, the computational complexity of building a big random forest (with 2000 trees) is dramatically higher than both Logistic Regression or Decision tree classifiers. The performance of decision tree with respect to recall is slightly better than Random Forest (0.45 vs 0.41). Notwithstanding the recall, its F-measure is drastically lower than that of Random Forest (0.24 vs 0.16) because of noticeably lower precision compared to Random Forest (0.10 vs 0.17, over 70% lower). This considerable improvement over a single decision tree was caused possibly through ensemble of decision trees by Random Forest and alleviating over fitting problem. Presumably the high uncertainty in data that potentially caused the single classifiers failed to generalise well, therefore, ensemble learning by Random Forest helped enhance the precision by reducing the errors over many sub-classifiers.

The Random forest classifier employed in this study was tweaked using the Hyper parameter optimisation integrated in MatLab 2017a. We have not observed any better results through manual parameter settings over that library which is computationally extensive. In this context, in practice, finding near-optimal random forest over all data is computationally very extensive, hence, some independent experiments were conducted on small proportion of data to set the parameter settings (Hyper parameter optimisation results with grid search). The decision tree classifier was also tweaked in the same way (with other Hyper Parameter Optimisation Results + Grid Search/Random Search). In particular, the results of decision tree before and after parameter tuning is significantly different but the random forest were not that sensitive.

Rotation Forest is another ensemble learning-based method which is relatively newer than other classifiers proposed in 2016 (Rodriguez et al., 2006) with many successful application. It works by simply generating classifier ensembles based on feature extraction. It iteratively splits randomly the feature set into K subset (K is a parameter of the algorithm) and applies principal component analysis (PCA) on each of which. Its key idea was to “to encourage simultaneously individual accuracy and diversity within the ensemble”; thus, to possibly alleviate the overfitting problem we can see in training of other ensemble-based algorithms such as Random Forest. Hence, one may expect higher performance than Random Forest classifier. Nevertheless, an experimental study by Bagnall et al. observed that rotation forest works better for problems with all continuous features (Bagnall et al., 2018). That is consistent with our experiment too; as possibly, the lower performance of Rotation Forest compared to Random Forest is associated with the fact that our feature space is not entirely continuous. This is an extremely important fact since many classifiers could not perform well over mixed feature space. The performance of Rotation Forest did not increase with increase in K, but similar to other classifiers, the recall significantly was improved from 0.51 to 0.75; whilst because of markedly lower precision, the F-measure decreased sharply about 0.05 from 0.19 to 0.14 for K = 10 and K = 50, respectively.

Overall, Logistic Regression and Random forest were the top performing classifiers with 0.23 and 0.24 F-measures. Furthermore, considering performance of classifiers with different parameter settings, it can be roughly expected that F-measure of about 0.20 is possibly an upper boundary that most classifiers can reach to through more or less tweaking. Notwithstanding reaching out to F-measure of over the upper limit seems to be associated with the capability of the classifier to better learn real patterns in training samples and generalise. In this context, it could conclude that Random forest obtained the best performance and trained the most practical model with 0.24 F-measure and a relatively good recall of 0.41. Logistic Regression stands just right to Random Forest with slightly lower F-measure. Despite that slight difference, its model is not practical for our application since both of its precision and recall are almost in the lowest quartile (both below 0.29). Meanwhile, interestingly, the SVM classifier with Polynomial kernel function is the only classifier with F-measure of 20 with a recall figure over 0.50. All of the other high F-measure models are coming with recall < 0.42. taking all into account, it could be said that SVM model is actually the second-best performing classifier with respect to both F-measure and recall.

Meanwhile, the performance of the proposed method is detailed in Table 4. Despite other classifiers, for training SDAE which is simply a neural network, the training samples cannot be fed into the network in a single step. Data should be fed into the model in small parts called batch. The batch containing 64 samples was utilised in our experiments as similarly was adopted primarily in Adam optimiser (Kingma & Ba, 2015) and suggested by other works (Jamei et al., 2017). There are other parameters such as sparsity weight, learning rate and L-2 regularisation parameters which were not altered from default values (set out already in Matlab with their default suggested parameters). The features extracted from SDAE were employed to train different classifiers independently. Actually, each classifier performed learning upon the feature space having either 16 or 32 extracted features. There are numerous combinations of parameters to tweak, notwithstanding these were among the best performing we have found that reveals general trend among candidate classifiers and the SDAE with integrated Soft Max layer.

**Table 4.** Performance of SDAE in combination of candidate classification algorithm Numbers in parentheses represent either the number of extracted features by SDAE, or the number of features in the last layer of SDAE.

Method	Measures			
	Precision	Recall	F1-Score	AUC
SDAE (16) + Random Forest	0.095	0.743	0.168	0.568
SDAE (32) + Random Forest	0.125	0.661	0.210	0.603
SDAE (16) + Logistic Regression	0.223	0.405	**0.288**	**0.704**
SDAE (32) + Logistic Regression	0.162	0.482	0.242	0.641
SDAE (16) + SVM (polynomial 3)	0.087	0.821	0.157	0.559
SDAE (32) + SVM (polynomial 3)	0.143	0.492	0.222	0.593
SDAE-Softmax (16)	0.188	0.601	**0.286**	**0.696**
SDAE-Softmax (32)	0.160	0.655	0.257	0.667

It is interesting to note that the Logistic Regression and Random forest that were among the top performing classifiers with 0.23 and 0.24 F-measures; when merged with SDAE performed quite differently. The Random Forest classifier performance measures are different in many ways over the extracted features of SDAE than the original feature space. This observation was pretty much expected considering an utter continuous feature space generated by SDAE. The F-measure obtained by Random Forest this time is noticeably lower with about 15% decrease (or 0.40 from 0.247 down to 0.210). As aforementioned, a key advantage of Decision Tree-based approaches about their capability to work with intact feature space; without the need for encoding nominal features and transformation that is typically mandatory for other classifiers to operate. Therefore, the SADE with RF classifiers did not perform as good as the RF alone. In spite of marginally lower F-measure, it seems this model is more practical with markedly higher recall (0.25 higher according to recall of 0.66 against 0.41 for SADE+RF and RF, respectively) whilst actually sacrificing about 0.05 of the previously obtained precision (0.12 vs 0.17). Despite Random Forest, the performance of Logistic Regression has improved remarkably with rising F-measure from 0.23 to 0.27 that is actually a big improvement. The quality of predictive model produced by logistic regression is also remarkably higher with the highest observed AUC of 70% against no greater than 60% for random forest.

It is worth noting that these models can be sorted on the ground of AUC values which shows how well a classifier predicts a value better than a random guess classifier. A random guess classifier can obviously acquire the AUC of 0.5% or 50%. In this context, we can see that only the proposed method with logistic regression and softmax can produce models with AUC of 0.70 that are acceptable in real application. The worst case we see AUC 0.64 and 0.66 for logistic regression and softmax, respectively. Meanwhile, the logistic regression obtained the highest AUC and highest precision whilst the softmax comes very close in AUC whilst generating an equivalent quality model with noticeably higher recall.

Furthermore, there is no general trend saying the lower number of features had a significant impact of the results. However, one can say, the top performing models performed nearly 4–8% better compared to their performance with 32 features having AUCs of 0.69 vs 0.66 for softmax and AUCs of 0.70 vs 0.64 for logistic regression models. This observation is followed by random forest models with 0.60 and 0.56 for 16 and 32-feature models, respectively. Despite that, for other classifiers such as SVM, this rule is reversed observing AUCs of 0.55 vs 0.59 for 16 and 32-feature models, respectively.

Moreover, it is critical to leverage the intervention strategy into model selection. For example, if the intervention strategy is by texting people, then the model with higher recall is obviously more preferable than higher precision. In this context, it is easy and much feasible to choose among models mostly when their AUCs are similar. Otherwise, it would not be easy comparing recall and precisions and selecting model based on which. For example, it is evident that very low-performing models over the testing sample that are actually Random forest and SVM produced unrealistically high recalls of 0.82 and 0.74 whilst their AUCs are about 0.55 signifying that these models do perform a little better than a random guess classifier. In this regard, among the top-forming models obtained, the SDAE with Softmax is more suitable taking into account the fact that we are interested in texting strategy for intervention. Despite that, if the intervention was through calling people which costs much, then undeniably the models with high precision are suitable considering which none of the produced models are good enough for such intervention.

Moreover, considering interpretation and studding causal relations, these series of models and in particular the deep learning models have inherently poor interpretability. Large parameter space and the interaction between neurons prevent us from interpreting the model coefficients directly. However, there are some approximation methods to roughly see which group of variables is relatively more important. Among various methods that can be adopted within machine learning application, some representative wrapper approaches are described expansively in (Hall & Holmes, 2003; Lazar et al., 2012) for identifying subset of more important features. In this study, the Representation Erasure (Li et al., 2016) is employed. Representation erasure is a general method for analysing and interpreting decisions made by a black-box model. We erase individual variables and observe how the model performance degenerates. If the model’s AUC decreases by a significant amount when we remove a particular variable, the model considers the variable to be important. In practice, due to the high number of variables and complexity of dimensions, we remove variables in groups of “Demographic information”, ’Appointment characteristics’, “Patient appointment history”, “Deprivation”, “Weather” and “Admission history” as mentioned in table 1. We then calculate the importance score for each variable group as the differences between the original deep learning model’s AUC and the same model but with the erased input.

**Figure 5.**
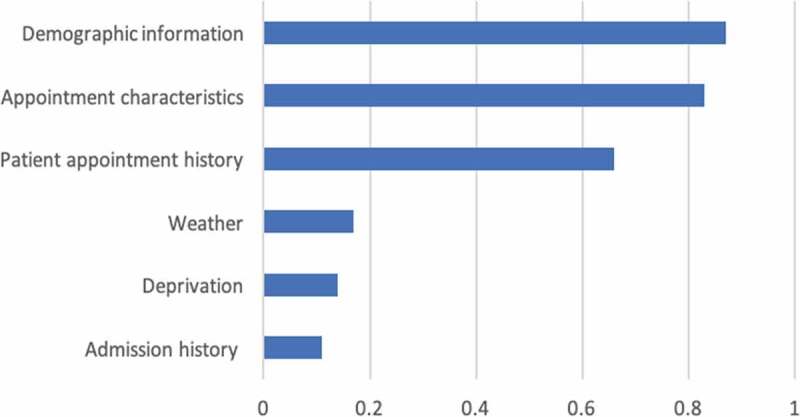
Variable group importance in attendance prediction.

The results are graphically represented in Figure 5, where the predictors are listed in descending order of importance. The results show “demographic information” as the most critical variable group that affect patients’ attendance, which includes patients’ age, ethnicity, gender, long-term condition, and address stability is important to affect attendance. “Appointment characteristics” is the second most important variable group that affects attendances outcomes, which includes speciality, treatment function, site, date/time, duration, follow-up or first-time appointment. This means that if only those variable groups are excluded from the model, there will be a more negative effect on the predictive capability of the model than the exclusion of other variable groups. Furthermore, patient appointment history is another important variable group that indicates attendance, which includes number/ratio of previously missed appointments, number/ratio of previously attended appointments, history of rebooking and cancellation and history of cancellation and rebook by the hospital. Weather, deprivation and admission history variable groups have less influence on attendance. From the managerial point of view, these insights can support the hospital operation team to provide appropriate support for patients to attend the appointment. For example, as the site of appointment is one of the important variables, we have started the collaboration with public transportation company to provide patients who live far away with a ticket voucher for their travel.

### 4.4. Risk profile visualisation with live patients’ data

**Figure 6.**
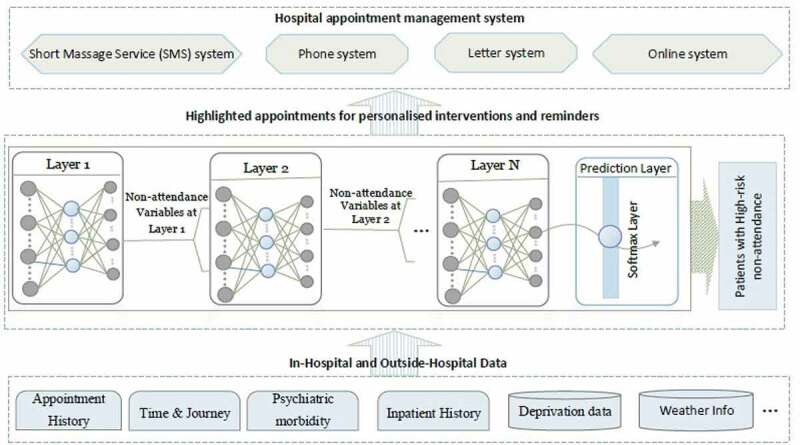
Non-attendance prediction model integrated with the hospital appointment system.

**Figure 7.**
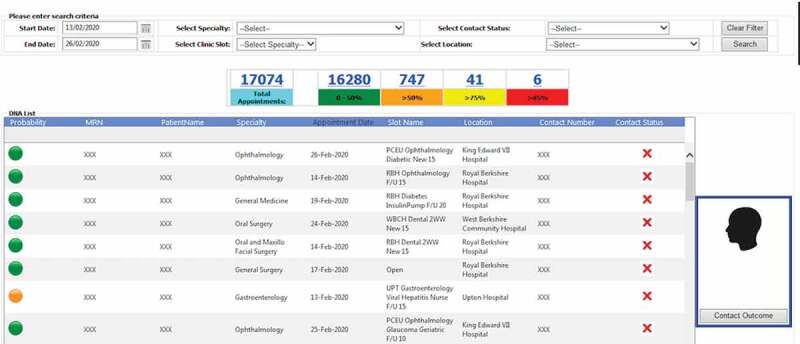
Non-attendance application in hospital reporting system (with identifiable information removed).

**Figure 8.**
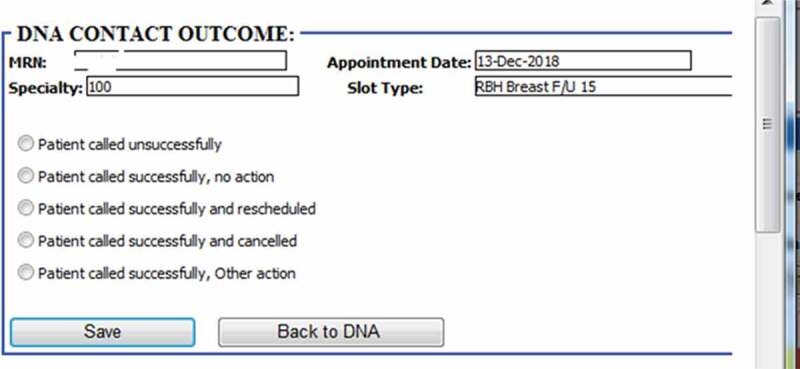
Contact actions (DNA refers to “Do not attend”).

One extremely important fact that districts this study from many others, although is yet in the experiment stage, is the fact that this has been successfully deployed our model into hospital business intelligence and reporting system as graphically shown in Figures 6,7 and 8. After deployment of the model, the obtained risk profiles of live patients’ data are visualised to hospital operational team for targeted intervention. As Figure 6 reveals the different layers from data layer at the bottom, prediction model at the middle and finally action layer which works based upon the recommendation of the model. Our research has been integrated with hospital information systems as automated algorithms into appointment systems. We have built a dataflow that actually fetches and process live data, feeds the transformed data into the model and employs the predicted risk into an outpatient appointment data table. After making prediction through the trained model in the middle layer, another team works at last level to take proper interventions. Figure 7 demonstrates how each patient’ non-attendance risk profile is visualised in hospital reporting system with identifiable information removed. Appointments at certain time period (e.g., appointments in next two weeks), speciality (e.g., general surgery), clinical slot (e.g., Breast F/U15) with different contact status (e.g., patients not contacted for appointment reminder) can be filtered (Figure 8) and accordingly visualised with predicted risk both individually and in the different risk groups. The risk profiles are defined based on the prediction probability produced by the model at appointment level for each patient. The high-risk patients (non-attendance risk over 80%) are flagged with red, middle-high risk patients (non-attendance risk between 60%-80%) with yellow, moderate risk patients (non-attendance risk between 50% and 60%) with orange and low-risk patients (non-attendance risk below 40%).

According to the risk profile, the hospital operational team will be able to prioritise the interventions methods. There are several interventions already implemented attempting to reduce non-attendance rates in hospitals. This includes reminder letter systems, phone reminder system (Shahriar Tavakoli-Tabasi, 2015) and short message services (SMS) reminder system (Guy et al., 2012; Taylor et al., 2012). Research shows that there is no significant reduction in non-attendance rates using reminder letter but there is evidence that telephone or text message reminder substantially reduce missed appointments (Gurol‐Urganci et al., 2013). In our case study SMS was the primary approach to remind patients about their appointments before the prediction model deployment. According to the risk profile, hospital operation team contact low- and middle-risk patients through message reminders. The high-risk patient will be contacted through both message and telephone as having conversations could be more effective with the ability of understanding patients’ potential reason and difficulties of missing the appointment so support could be provided for those patients. As shown in Figure 8, five contact outcomes are recorded to track how those interventions affect patient’s behaviours regarding planning and attending the appointments. For outcomes of other actions, it involves potential support available according to patient’s situations (e.g., transportation support). Those contact outcome and action data can be further used to analyse the effectiveness of the interventions as well as to update the model over time with new appointments data.

## 5. Conclusions and future works

In this study, we represented a novel non-attendance prediction method incorporating a broad spectrum of factors relating to health, social economics and environment for improved understanding and prediction of patient behaviours. The proposed approach is an end-to-end deep learning model which adopted the latest architecture of sparse stacked denoising autoencoders (SDAEs). The SDAEs were used for data reconstruction, dimensionality reduction and classification. It was used also as a hybrid method with other classifiers. In the prediction phase, a softmax layer that has been used in modern deep learning models was added to the network. This layer produced the probability of non-attendance events based on the outputs of the last hidden unit in SDAE. The performance of the model over the testing samples was compared with other classification models which revealed that the logistic regression, and softmax classifiers could produce high-quality models with AUCs around 0.70. The experiments illustrated that the proposed approach outperformed other approaches regarding important evaluation metrics including AUC-ROC, Precision, Recall, and F-Score.

An important advantage of this model is its capability to represent complex datasets with high dimensionality and sometimes incomplete information, which is widespread in real-world practical application. One critical benefit of our proposed approach is the scalability. Scalability is defined in three different ways: (1) the number of variables and (2) the number of samples we can use and most importantly (3) model update over time. We could add new variables to the existing model with the same practice. New variables provide a way to incorporate more information into the model resulting in a more reliable model for managers. It is a commonplace that every practical application has a life-period. In this context, update-&-upgrade potential is a critical issue which impacts the future of organisations by directly leveraging the operational costs. Artificial intelligence, fortunately, produces a highly scalable application that is easy to maintain and easy to upgrade. For example, considering our application in two years later, we could re-train the model, add or remove any variables, incorporate the knowledge of latest patients’ records, and ultimately achieve an updated model with higher performance and reliability.

Another bottleneck to address as future work is the problem of fine-tuning procedures and dealing with several free parameters which is quite challenging. Perhaps in future with advancing AI technology, we would see high-scale self-adaptable algorithms. From another viewpoint, more relevant data and higher quality improve the performance of all current models. We believe the current trends for developing health-care systems in the world follow strategies to reduce operational costs, reduce clinical costs, and improve clinical outcomes. Adopting such intelligent algorithms in healthcare application with high-scale dimension could potentially contribute to this process.

## Appendix A

**Table:** Distribution of non-attendance across care specialities and other variables.

Attribute name	Category/value	Non-attendance	% Non-Attendance	# Appointments	% Appointments
**Ethnicity**	African	4350	1.46%	37,805	1.01%
	Any other Asian background	8509	2.85%	91,227	2.43%
	Any other Black background	2208	0.74%	18,991	0.51%
	Any other ethnic group	5369	1.80%	54,456	1.45%
	Any other mixed background	1797	0.60%	16,816	0.45%
	Any other white background	17,815	5.96%	201,197	5.37%
	Bangladeshi	670	0.22%	6887	0.18%
	British	186,419	62.39%	2,556,810	68.2%
	Caribbean	3168	1.06%	30,095	0.80%
	Chinese	963	0.32%	13,398	0.36%
	Indian	6060	2.03%	68,277	1.82%
	Irish	1228	0.41%	15,282	0.41%
	Not known	3531	1.18%	9201	0.25%
	Not stated	45,502	15.23%	540,797	14.4%
	Pakistani	7168	2.40%	62,762	1.67%
	White and Asian	626	0.21%	6502	0.17%
	White and Black African	392	0.13%	3503	0.09%
	White and Black Caribbean	1162	0.39%	9165	0.24%
	Others	1875	0.63%	4114	0.11%
**Care Group Description**	CG2 – Planned	157,311	52.65%	1,972,086	52.6%
	CG3 – Networked	79,817	26.71%	1,056,996	28.21%
	CG1 – Urgent	56,686	18.97%	702,491	18.75%
	NULL	4998	1.67%	15,712	0.42%
**Speciality**	Accident and Emergency	477	0.002%	4732	0.001%
	Allied Health Professional Episode	18,811	0.063%	268,796	0.071%
	Anaesthetics	6804	0.023%	87,880	0.023%
	Audiological Medicine	11,759	0.039%	213,210	0.056%
	Cardiology	8968	0.030%	167,115	0.044%
	Chemical Pathology	283	0.001%	1898	0.0005%
	Clinical Haematology	7740	0.026%	67,392	0.017%
	Clinical Oncology	4726	0.016%	101,404	0.027%
	Clinical Physiology	0	~0	8	~0
	Community Medicine	0	~0	2	~0
	Critical Care Medicine	327	0.001%	9909	0.0026%
	Dental Medicine Specialities	1	0.000%	10	~0
	Dermatology	10,573	0.035%	181,812	0.048%
	Endocrinology	8103	0.027%	60,910	0.016%
	ENT	14,710	0.049%	160,874	0.043%
	Gastroenterology	8533	0.029%	89,036	0.024%
	General Medicine	1322	0.004%	12,534	0.003%
	General Surgery	11,871	0.040%	183,527	0.048%
	Genito-Urinary Medicine	213	0.001%	1835	0.0004%
	Geriatric Medicine	1098	0.004%	11,362	0.003%
	Gynaecology	6547	0.022%	116,086	0.031%
	Haematology	60	~0	635	0.0002%
	Medical Oncology	69	~0	1521	0.0004%
	Midwife Episode	9681	0.032%	61,306	0.0164%
	Nephrology	4278	0.014%	56,974	0.015%
	Neurology	6971	0.023%	72,550	0.019%
	Neurosurgery	0	~0	2	~0
	Nursing Episode	38	0.032%	65	~0
	Obstetrics	9470	~0	128,421	0.034%
	Obstetrics and Gynaecology	17	0.215%	197	~0
	Ophthalmology	64,260	0.008%	730,747	0.195%
	Oral and Maxilla Facial Surgery	2457	0.013%	23,099	0.006%
	Oral Surgery	3960	0.006%	55,880	0.015%
	Orthodontics	1783	0.001%	20,289	0.005%
	Paediatric Cardiology	255	~0	3627	0.001%
	Paediatric Surgery	61	0.043%	471	0.0001%
	Paediatrics	12,728	~0	115,615	0.031%
	Palliative Medicine	3	0.005%	7	~0
	Plastic Surgery	1538	~0	27,379	0.007%
	Psychotherapy	11	~0	352	~0
	Radiology	107	0.004%	1232	0.0003%
	Rehabilitation	1138	0.027%	11,931	0.003%
	Respiratory Medicine	8109	0.025%	94,251	0.025%
	Rheumatology	7403	0.075%	94,660	0.025%
	Trauma and Orthopaedics	22,281	0.015%	344,663	0.092%
	Unknown	4597	0.048%	13,726	0.004%
	Urology	14,257	0.001%	145,304	0.039%
	Others	414	0.002%	2049	0.0005%
**Gender**	Female	165,373	55.40%	2,160,125	57.65%
	Male	133,419	44.69%	1,586,900	42.35%
**Multiple deprivation indexes**	Average score of multiple deprivation in for patients with non-attendance = 14.81860624		Average rank of multiple deprivation indexes for patients with non-attendance = 20,780.94586		

^a^“~0” indicates the numbers close to zero, “#” denotes for “the number of”.
